# An outbreak of *Pseudomonas aeruginosa* endophthalmitis following cataract surgery: a case series and lessons learned

**DOI:** 10.1093/jscr/rjaf069

**Published:** 2025-02-27

**Authors:** Abdulmajeed Al Khathami, Abdullah I Abbas, Amani AlGhramah, Wejdan S Alghamdi, Abdullah N AlOlyani, Khaled A A Ghaith

**Affiliations:** Department of Ophthalmology, King Fahad Hospital, Al Baha Health Cluster, PO Box 65511, Al Baha, Saudi Arabia; Department of Ophthalmology, King Fahad Hospital, Al Baha Health Cluster, PO Box 65511, Al Baha, Saudi Arabia; Department of Optometry, Prince Mishary Bin Saud Hospital, Al Baha Health Cluster, PO Box 65511, Al Baha, Saudi Arabia; Faculty of Medicine, AlBaha University, PO Box 65511, Al Baha, Saudi Arabia; Faculty of Medicine, AlBaha University, PO Box 65511, Al Baha, Saudi Arabia; Department of Ophthalmology, King Fahad Hospital, Al Baha Health Cluster, PO Box 65511, Al Baha, Saudi Arabia

**Keywords:** postoperative endophthalmitis, cataract surgery complications, infection control, *pseudomonas aeruginosa*

## Abstract

Endophthalmitis is a severe intraocular infection that can cause substantial visual impairment or blindness. Although it is an uncommon post-cataract surgery complication. In this case series, we present a group of patients who underwent surgery on the same day by the same surgeon. Our goal is to investigate the causes of this outbreak, explore the management strategies employed, and derive lessons learned to prevent future occurrences. This case series presents five immunocompetent patients who underwent uncomplicated cataract surgery at a single center and developed acute postoperative endophthalmitis. All patients presented with severe eye pain and decreased vision on the 2nd day post-surgery; one patient presented 15 days later. Following their surgeries, they were prescribed prednisone acetate and moxifloxacin, both to be taken every 2 h. Their visual acuity at presentation was light perception. Immediate vitreous tap and administration of intravitreal antibiotics. Despite these urgent interventions, the outcomes varied among the patients. In the short term, no significant improvement in visual acuity was noted; all patients continued to experience severely limited vision. The long-term consequences were grave: three of the five patients eventually underwent evisceration due to the severity of the infection and persistent inflammation. This underscores the aggressive nature of the infection and the challenges in managing such severe cases of endophthalmitis. This case series underscores the critical need for rigorous infection control protocols. By meticulously addressing these challenges, healthcare providers can enhance patient outcomes and significantly reduce the risk of future outbreaks in ophthalmic surgical settings.

## Introduction

Cataract surgery is one of the most common ophthalmic procedures performed worldwide, with millions of patients undergoing this surgery each year [1]. Generally recognized as safe and effective, the procedure does carry certain risks, among which endophthalmitis stands out as a severe and potentially blinding intraocular infection. This complication, although rare with an incidence rate of 0.07%–0.12% [1], endophthalmitis following paras plana vitrectomy (PPV) is relatively uncommon. The incidence ranges between 0.03% and 0.14% for 20 G PPV [2]. Post-injection endophthalmitis, the incidence is between 0.028%–0.056% and does not appear to vary based on geographic location [3]. Endophthalmitis results from the intraocular spread of external microorganisms, with potential sources including intraocular solutions, the surgical environment, phacoemulsification equipment, surgical instruments, intraocular lenses, and viscoelastic agents [4–7]. The incidence rates of endophthalmitis vary significantly by region, reflecting differences in surgical practices and infection control standards. For instance, a study in Saudi Arabia reported a lower incidence rate of 0.08%, which, while below the rates observed in the UK (0.16%) and Canada (0.15%), remains higher than those reported in Singapore (0.04%), Sweden (0.03%), and the USA (0.025) [8, 9]. This case series addresses a cluster of five endophthalmitis cases following cataract surgery at a private medical center in Al Baha, Saudi Arabia. This paper will exploring contributing factors to management strategies and emphasize the crucial role of strict adherence to infection control protocols in ophthalmic surgical settings.

## Case presentation

This series examines five immunocompetent patients who developed acute postoperative endophthalmitis after undergoing cataract surgery at a single center, emphasizing the severity and management of each case.

**Figure 1:**
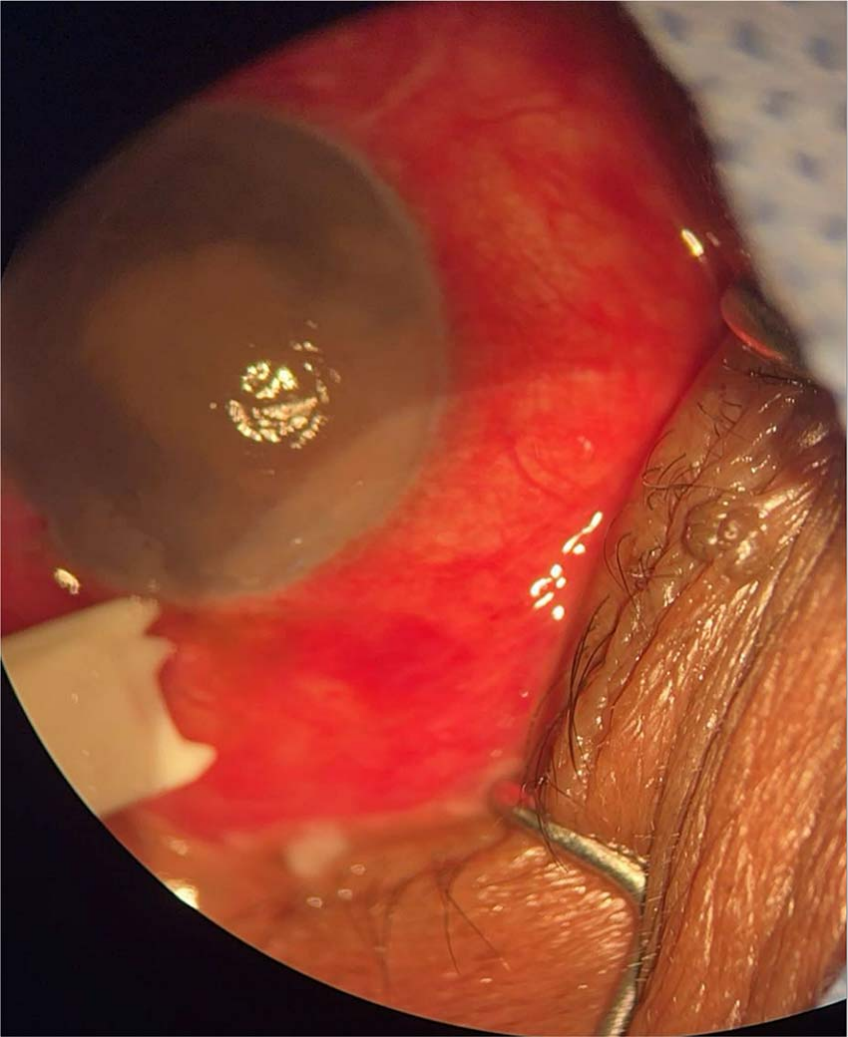
Surgical microscope examination showing hypopyon, chemosis, and conjunctival injection in patient 1.

**Figure 2:**
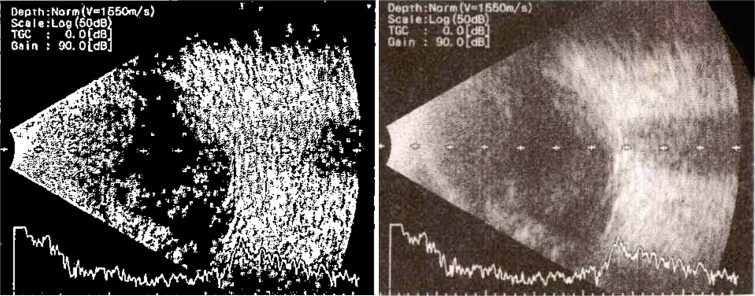
B-scan ultrasound demonstrating mild to moderate vitritis in endophthalmitis case in patient 1.

**Patient 1**: A 66-year-old Sudanese male presented to the emergency room with severe pain in his right eye 2 days post-phacoemulsification. Examination revealed only light perception, elevated intraocular pressure (IOP), a hazy cornea, hypopyon, and extensive pupillary membrane (Fig. 1). A B-scan ultrasound (Fig. 2). He underwent a vitreous tap and received intravitreal antibiotics.

**Figure 3:**
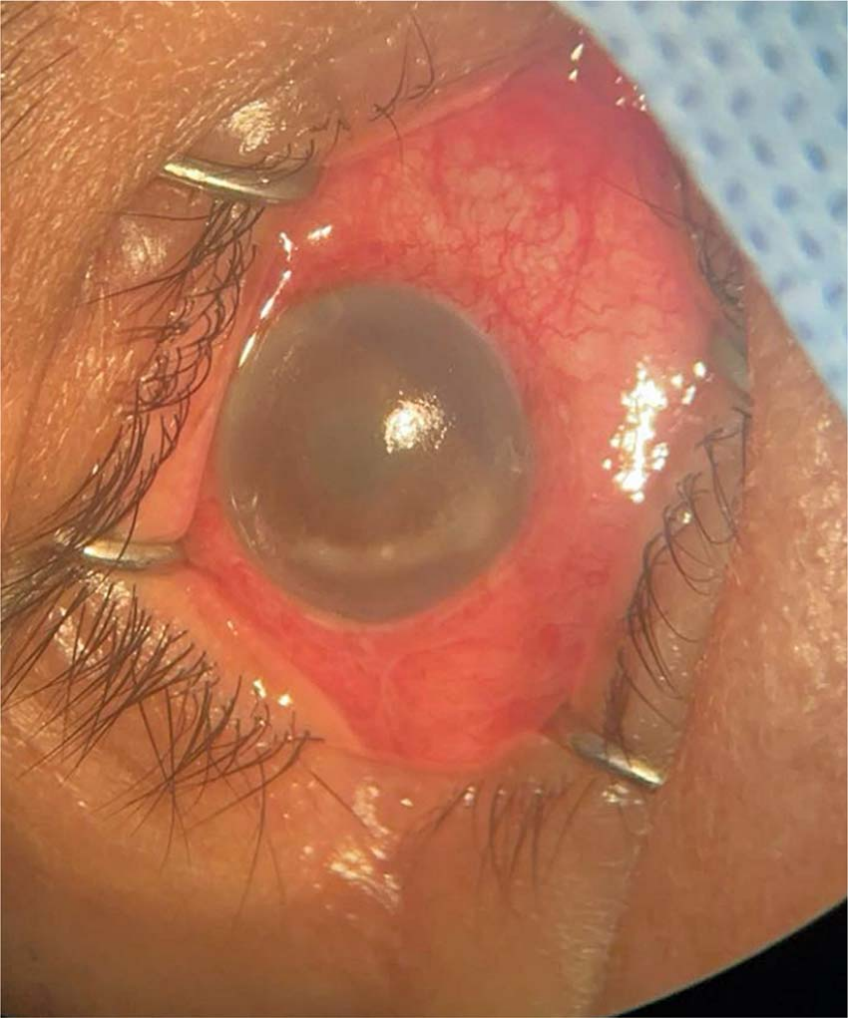
Surgical microscope examination showing hypopyon, chemosis, ciliary, and conjunctival injection in patient 2.

**Figure 4:**
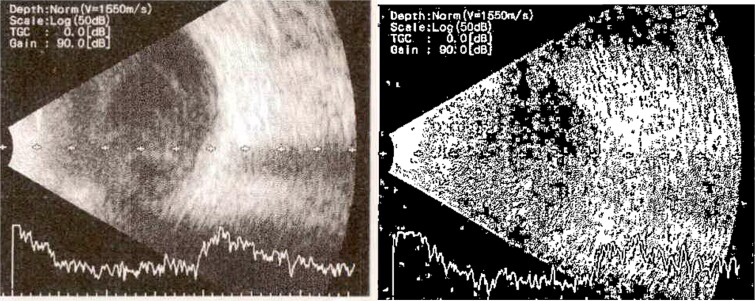
B-scan ultrasound showing moderate vitreous echoes and vitreous abscess in patient 2.

**Patient 2**: A 69-year-old Saudi female experienced severe right eye pain after surgery on the same day as patient 1. Visual acuity was reduced to hand motions, and examination showed conjunctival ciliary injection, corneal edema, and hypopyon (Fig. 3). B-scan confirmed vitritis (Fig. 4). She received similar emergency interventions as patient 1.

**Figure 5:**
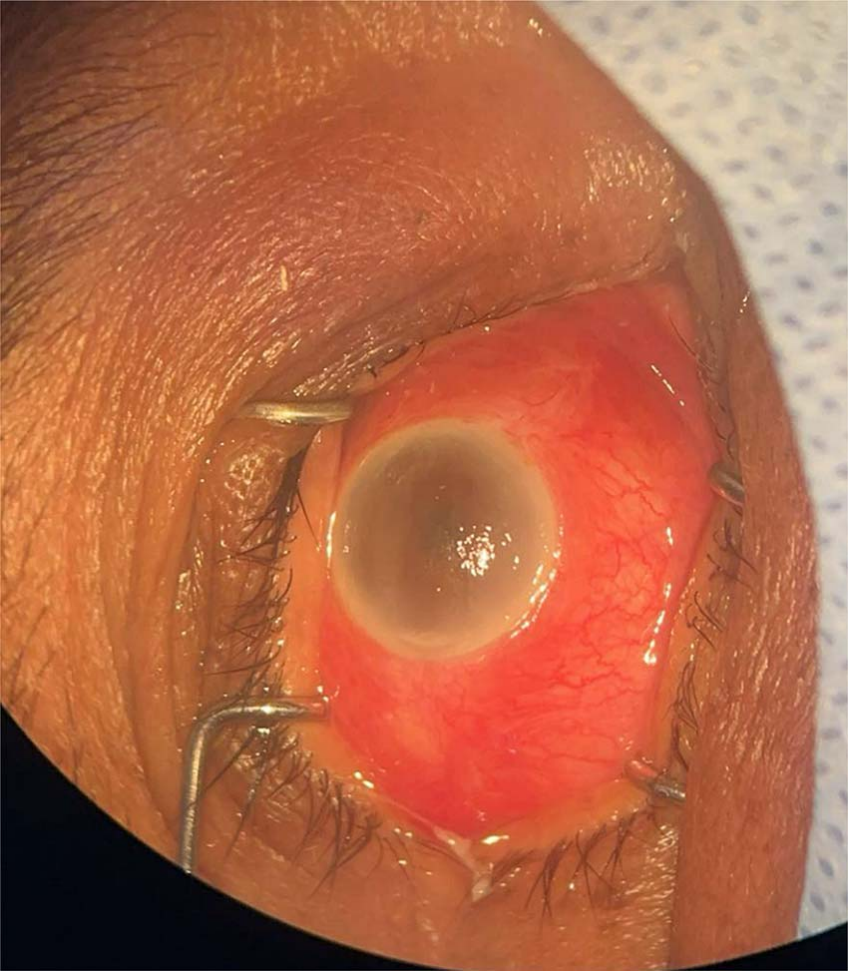
Surgical microscope examination showing hypopyon, chemosis, ciliary, and conjunctival injection in patient 3.

**Figure 6:**
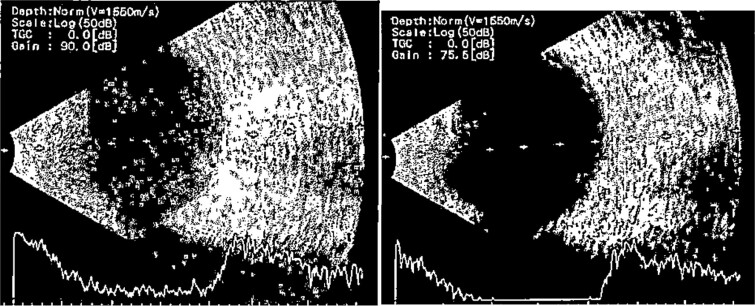
B-scan ultrasound demonstrating mild to moderate vitritis in endophthalmitis case for patient 3.

**Patient 3**: A 63-year-old Saudi female reported pain and decreased vision in her right eye. On examination 4 days post-surgery, her vision was PL. Examination findings included severe conjunctival injection (Fig. 5). B-scan (Fig. 6). Treatment included vitreous tap and intravitreal antibiotics.

**Figure 7:**
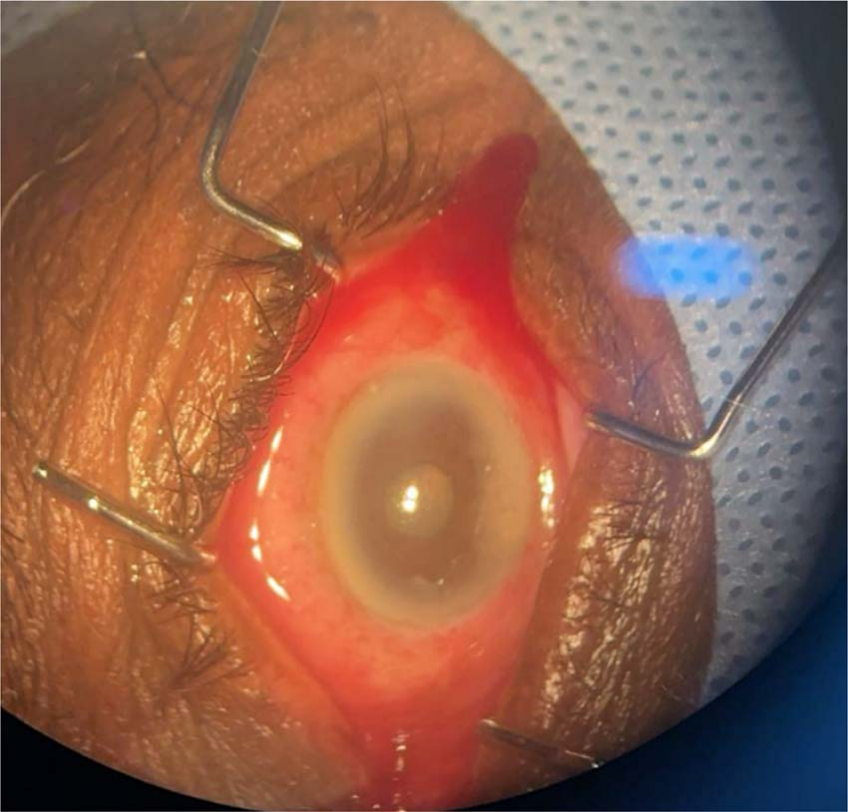
Surgical microscope examination showing hypopyon, chemosis, ciliary, and conjunctival injection in patient 4.

**Figure 8:**
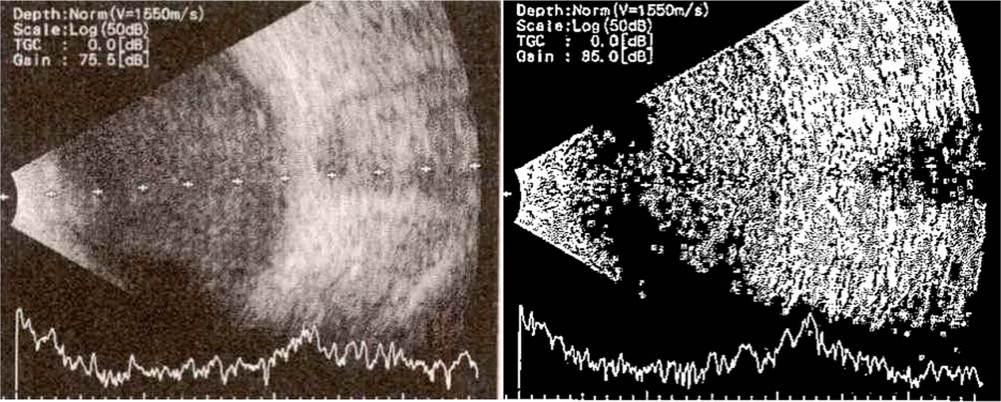
B-scan ultrasound demonstrating moderate vitreous echoes and vitreous abscess in patient 4.

**Patient 4**: A 74-year-old Sudanese male visited the emergency room with severe left eye pain and loss of vision. His visual acuity was PL; examination showed hypopyon (Fig. 7). B-scan demonstrated vitritis (Fig. 8). He underwent emergency vitreous tap and intravitreal antibiotic therapy.

**Figure 9:**
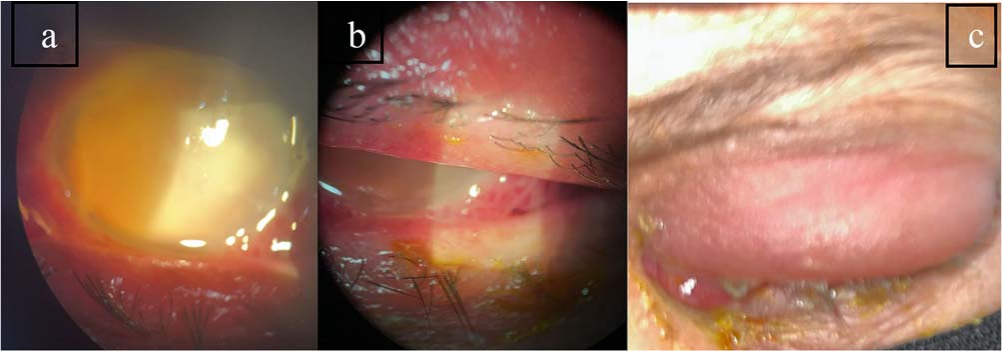
Slit lamp examination of patient 5 showing (a) corneal haziness, (b) severe chemosis and ciliary injection, and (c) severe redness and swelling of the left upper lid.

**Figure 10:**
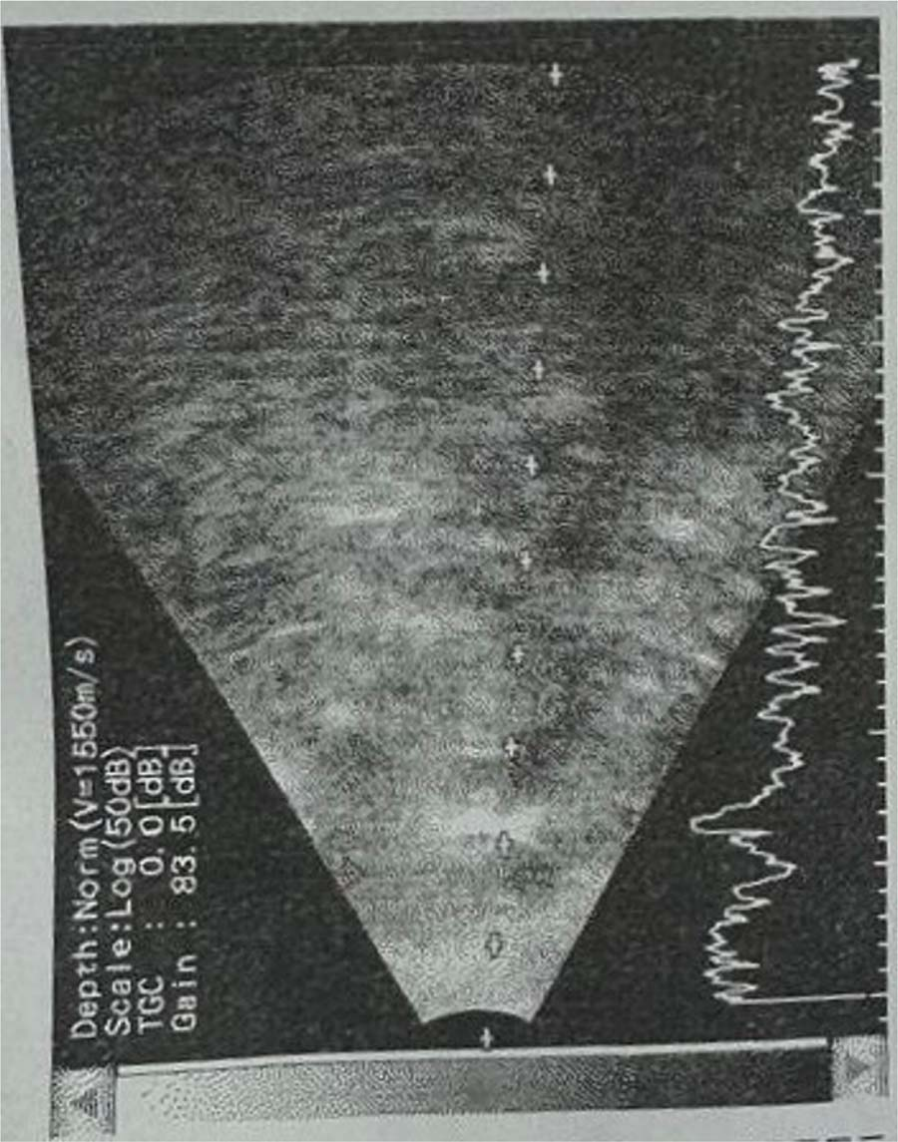
B-scan ultrasound showing sever vitreous echoes and vitreous abscess in patient 5.

**Figure 11:**
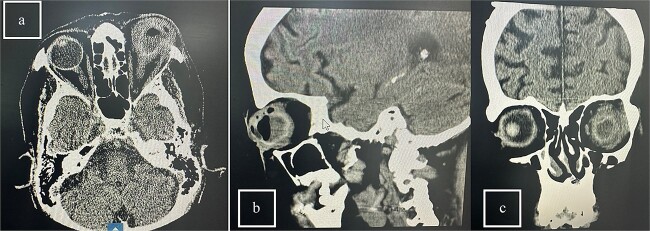
CT scans of the orbit for patient 5 showing (a) coronal view with dense fluid content and air-fluid leveling, (b) axial view with edematous changes in the intra-conal fat, and (c) sagittal view with involvement of the extra-ocular muscles.

**Patient 5**: Fifteen days post-phacoemulsification, a 65-year-old Saudi female presented with no light perception (NPL) and severe lid edema (Fig. 9). B-scan revealed vitritis (Fig. 9). Figure 10 showing the B-Scan Ultrasound, illustrates the findings in Patient 5. Computed tomography (CT) scans of the orbit showed pan ophthalmitis (Fig. 11). She received intravitreal antibiotics immediately, followed by a pars plana vitrectomy.

### Follow-up

After initial treatment with prednisone acetate and moxifloxacin every 2 h. Vitreous samples were collected from the patients within 30 min of presentation and subjected to microbiological analysis. Cultures were grown on blood agar, chocolate agar, and Sabouraud dextrose agar. Table 1 summarizes the patient characteristics, clinical features, and details of the operations and microbiological findings of each case.

**Table 1:** Summary of patient characteristics, clinical features, and operation details

**Patient**	**Age (Years)**	**Gender**	**Presenting visual acuity**	**Duration after surgery**	**Systemic diseases**	**Clinical features**	**Corneal involvement**	**Hypopyon**	**Fundus view**	**B-Scan findings**	**Primary surgical procedure**	**Secondary surgical procedure**	**Visual acuity after 3 weeks**	**Isolated organism**	**Antibiotic sensitivity**	**Systemic treatment**	**Follow-up visual acuity**
1	66	Male (Sudanian)	Perception of Light (PL), OD	2 days	None	Severe pain, high IOP, massive pupillary membrane	Hazy cornea, ciliary injection	Yes	No View	Vitritis, flat retina	Vitreous tap, intravitreal antibiotics	Pars plana vitrectomy	Count finger OD, 20/30 OS	*P. aeruginosa*	Sensitive to: Amikacin, Cefepime, Ceftazidime, Ciprofloxacin, Gentamicin, Imipenem, Levofloxacin, Meropenem, Tazocin	IV Vancomycin, Ceftazidime	Count Fingers (CF)
2	69	Female	Hand Motion (HM), OD	2 days	None	Severe right eye pain	Mild corneal edema, shallow AC, severe ciliary injection	Yes	No View	Vitritis, flat retina	Vitreous tap, intravitreal antibiotics	Pars plana vitrectomy	PL OD, 20/60 OS	*P. aeruginosa*	Sensitive to: Amikacin, Cefepime, Ceftazidime, Ciprofloxacin, Gentamicin, Imipenem, Levofloxacin, Meropenem, Tazocin	IV Vancomycin, Ceftazidime	No Light Perception (NPL)
3	63	Female	Perception of Light (PL), OD	2 days	Hypertensive	Severe eye pain, vision diminishment	Severe conjunctival injection, chemosis, corneal haze	Yes	No View	Mild vitritis	Vitreous tap, intravitreal antibiotics	Pars plana vitrectomy + Evisceration	-	*P. aeruginosa*	Sensitive to: Amikacin, Cefepime, Ceftazidime, Ciprofloxacin, Gentamicin, Imipenem, Levofloxacin, Meropenem, Tazocin	IV Vancomycin, Ceftazidime	Evacerated
4	74	Male (Sudanian)	Perception of Light (PL), OS	2 days	Diabetic	Severe pain with loss of vision	Pupillary membrane	Yes	No View	Vitritis	Vitreous tap, intravitreal antibiotics	Pars plana vitrectomy + Evisceration	-	*P. aeruginosa*	Sensitive to: Amikacin, Cefepime, Ceftazidime, Ciprofloxacin, Gentamicin, Imipenem, Levofloxacin, Meropenem, Tazocin	IV Vancomycin, Ceftazidime	Evacerated
5	65	Female	No Light Perception (NPL)	6 days	Diabetic	Severe pain, discharge, lid swelling, loss of vision, corneal haziness	Severe conjunctival injection, corneal haze	No	No View	Vitritis	Vitreous tap, intravitreal antibiotics	Pars plana vitrectomy + evisceration	-	*P. aeruginosa*	Sensitive to: Amikacin, Cefepime, Ceftazidime, Ciprofloxacin, Gentamicin, Imipenem, Levofloxacin, Meropenem, Tazocin	IV Vancomycin, Ceftazidime + topical treatment, 1 g Vancomycin, Ceftazidime Q12H	Evacerated

### Follow-up and outcome

Despite aggressive initial management, the prognosis for most patients was poor. The final visual acuity outcomes varied from evisceration to merely counting fingers, indicating severe and enduring impacts on their vision.

**Figure 12:**
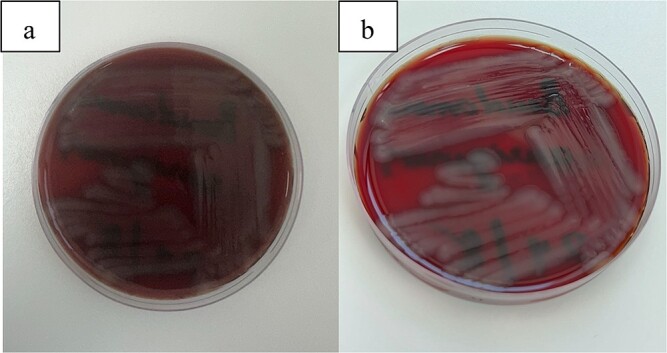
(a, b) This figure illustrates the characteristic growth of *Pseudomonas aeruginosa* on blood agar plates.

Vitreous cultures demonstrated characteristic growth of *Pseudomonas aeruginosa* on blood agar plates (Fig. 12). Comprehensive health surveillance was conducted within the hospital, particularly targeting the surgical environment. This investigation identified *Sphingomonas paucimobilis*, a different microbial agent from the initial culture results.

## Discussion

Endophthalmitis remains a rare yet severe complication of cataract surgery, with incidence rates estimated between 0.07% and 0.12% [1]. The occurrence of endophthalmitis can be particularly detrimental, not only affecting patients but also impacting the professional standing of surgeons and medical facilities through reputational damage and potential legal repercussions, such as the revocation of medical licenses.

In this series, we detail a troubling outbreak of endophthalmitis at a private medical center in Al Baha, Saudi Arabia, characterized by the rapid emergence of four cases shortly after surgical procedures. This pattern suggests a probable common source of infection, likely attributable to breaches in aseptic protocols or failures in the sterilization process. The most common causes of the outbreaks were contaminated solutions, 10 reports (37%), and contaminated phacoemulsification machines, six reports (22.2%). Other possible sources of contamination included ventilation systems, three (11.1%); defective sterilization, three (11.1%); miscellaneous, three (11.1%); in five outbreaks (18.5%), no possible source could be identified. Bacteria occurred in 23 outbreaks (85.2%) and fungus in four (14.8%). *P. aeruginosa* was causative in 14 of 27 (51.8%) gram-negative bacteria [11,12].

*P. aeruginosa*, not typically part of the normal conjunctival or periocular flora, has been frequently identified in these outbreaks, implicating external contamination sources [6]. This bacterium’s role is exacerbated by its increasing resistance to multiple antibiotics, propelled by mechanisms such as efflux pump overexpression, reduced outer membrane permeability, metallo-beta-lactamase production, and alterations in topoisomerases critical for quinolone effectiveness [10]. The resilience and virulence of *P. aeruginosa*, capable of rapid proliferation within the ocular environment, present significant challenges in clinical settings, demanding heightened vigilance and stringent control measures to prevent and manage infections. Its capability to form biofilms and sustain chronic infections further complicates treatment strategies, often necessitating aggressive and multi-faceted therapeutic approaches [13].

Our investigation into the outbreak of endophthalmitis suggests that the contamination of surgical instruments or solutions likely contributed to the infection spread, underscoring the critical importance of stringent infection control practices. To mitigate the risk of such outbreaks and safeguard patient safety, several key strategies must be implemented: Firstly, strict adherence to proven sterilization techniques is essential. Aiming to achieve a significant reduction in the microbial load present in the surgical environment [14].

Secondly, implementing enhanced pre-operative screening protocols can aid in identifying patients who may be at higher risk of infection. These individuals may require additional preventative measures, which includes rigorous cleaning protocols, the use of effective air filtration systems, and the application of prophylactic treatments such as povidone-iodine antisepsis to reduce the risk of infection [15].

Education and training for all surgical staff on the best practices in infection prevention are also paramount [16]. Finally, robust surveillance and reporting systems are crucial. These systems should be designed to quickly identify and respond to any instances of infection, allowing for timely interventions that can significantly reduce the impact of outbreaks [17].

The management of endophthalmitis involves prompt intravitreal administration of antibiotics, supplemented by topical and systemic therapies. Despite these measures, the outcomes in our cases were largely poor, underscoring the need for early detection and rapid response to prevent serious visual impairment. The lessons from this outbreak stress the importance of comprehensive strategies that include both preventive measures and swift therapeutic responses to manage and mitigate the risks associated with endophthalmitis. Through rigorous analysis and adaptation of our infection control protocols, we aim to prevent future occurrences, ensuring the safety and well-being of our patients and upholding the standards of care expected in ophthalmic surgery.

## Conclusion

The endophthalmitis outbreak underscores the necessity for stringent infection control and diligent surgical practice adherence. Effective strategies, including rigorous sterilization, enhanced pre-operative screening, and staff education, are paramount. This case series highlights the critical need for swift detection and response to prevent severe complications and ensure patient safety. Continuous improvement in clinical protocols and surveillance is essential to mitigate future risks in ophthalmic surgeries.
